# Identifying the Prevalence, Trajectory, and Determinants of Psychological Distress in Extremity Sarcoma

**DOI:** 10.1155/2015/745163

**Published:** 2015-02-12

**Authors:** Melissa H. Tang, David J. Castle, Peter F. M. Choong

**Affiliations:** St. Vincent's Hospital Melbourne, 35 Victoria Parade, Fitzroy, VIC 3065, Australia

## Abstract

*Objective*. Extremity sarcoma (ES) is a rare cancer that presents with unique challenges. This study was performed to identify the prevalence, trajectory, and determinants of distress and characterise sources of stress in this cohort. *Methods*. Consecutive patients with ES were prospectively recruited between May 2011 and December 2012. Questionnaires were administered during initial diagnosis and then six months and one year after surgery. *Results*. Distress was reported by about a third of our cohort and associated with poorer physical function, poorer quality of life, and pain. In addition to fears regarding mortality and life role changes, the most common sources of stress were centered on dissatisfaction with the healthcare system, such as frustrations with a lack of communication with the hospital regarding appointments and lack of education regarding management and outcomes. *Conclusions*. Psychological distress presents early in the cancer journey and persists up to one year after surgery. Distress is associated with negative outcomes. Active screening and effective interventions are necessary to improve outcomes. Sources of stress have been identified that may be amenable to targeted interventions.

## 1. Introduction

Psychological distress in cancer is associated with nonadherence to treatment recommendations, poorer satisfaction with care, poorer interpersonal relationships (resulting in poorer quality of relationships with both formal and informal social support sources and healthcare professionals), utilization of ineffective coping strategies (e.g., helplessness, passive coping, and risk taking behaviours), and poorer overall quality of life (QoL) [1–3]. The impact of psychological distress on people with extremity sarcoma (ES) has not been extensively studied.

Because of the rarity of sarcomas, homogeneous studies with large sample sizes may be difficult to obtain. Previous early studies exploring psychological distress and QoL in ES patients tended to be limited to cross-sectional, retrospective studies of survivors [4–10]. Most had small numbers of participants and few were prospectively designed with a baseline measure of QoL and mental health or controlled for the context of the timepoint of the cancer journey [11–15]. Compared to other cancers' patients, ES patients may represent a unique cohort with additional considerations. Compared to other cancers, ES is rare and affects people across all age groups and is associated with more physical disability than other cancer types [16]. These factors suggest that sarcomas require unique study and results from studies of other cancers cannot simply be extrapolated to represent ES patients.

In a systematic review of papers from 1972 to 2002, Massie [17] found that depressive symptoms were prevalent in 9% to 58% of cancer patients, which was highly dependent on the site of the primary. Lung and pancreatic cancers displayed the highest levels of distress (up to 57.6% and 52.2%, resp.), brain, head, and neck cancers displayed high levels of distress (up to 48.6%), and breast and prostate cancers displayed moderate levels of distress (up to 35.4% and 30.5%, resp.) [18, 19]. Prevalence was associated with prognosis of the cancer, the morbidity associated with the cancer (especially physical function and pain), and the effect the cancer or its treatment had on physical appearance and body image. In sarcoma-specific cohorts, the prevalence of depression ranged from 13.7% to 33.3% and the prevalence of anxiety disorders ranged from 11.8% to 47.2% [5, 7, 8, 14, 15], whilst the prevalence of psychological distress has been identified to be as high as 77% in one study [9].

Everyone's cancer journey is different. The experience is a continuum based on various hallmarks, which may include diagnosis and staging of cancer; treatment (surgery and/or radiation and chemotherapy); recovery and rehabilitation; follow-up and surveillance; and recurrence or terminal phase [14]. In general, levels of psychological distress reduce with time, with peak distress experienced in the initial month to one year after diagnosis and treatment, and may persist for a period of time following treatment [20–24].

Risk factors for psychological distress in the psychooncology population include previous psychiatric distress [15, 17, 25–29], high level of function impairment [2, 26, 30–32], pain [17, 25, 33–36], lower socioeconomic status [2, 19, 29, 37, 38], female gender [17, 39], younger age [17, 18, 40–42], and poor perceived social support [26, 29, 32].

This study was performed to address the following aims: (1) characterize the sources of stress preoperatively in this cohort; (2) identify the prevalence and trajectory of distress in people with ES from before surgery to one year after surgery; (3) compare distressed versus nondistressed participants; and (4) identify the determinants of distress one year after surgery.

## 2. Methods

All patients from the Australian states of Victoria and Tasmania with newly diagnosed ES who were referred to the Victorian Sarcoma Service (VSS) between May 30, 2011, and December 31, 2012, were screened for suitability for inclusion. The VSS is a dedicated multidisciplinary team of healthcare professionals managing patients above the age of fifteen years with sarcoma in both public and private hospital settings. Patients were eligible if they were diagnosed with an ES and surgery was planned to be part of their management. Ethics approval was acquired at all the relevant institutions. Patients were approached after their consultation with their healthcare professional immediately following the diagnosis of the ES by a member of the research team and invited to participate. Privately insured patients were approached in the private rooms of their healthcare professional and publically funded patients were approached at the hospital outpatient clinics. Participants completed their questionnaires within a week of their initial contact and questionnaires were returned within two weeks of initial contact via a reply paid envelope. This represented the first timepoint, *t* = 0, which was the preoperative, diagnosis phase. The second timepoint, *t* = 1, and third timepoint, *t* = 2, were at six months and twelve months after surgery, respectively. Participants were followed up through verbal contact and posting of the health questionnaires and returned within two weeks via a reply paid envelope.

## 3. Measures

### 3.1. Demographic and Health-Related Information

Medical information was gathered and verified through record linkage with secure medical records and consultation with pathologists, oncologists, radiation oncologists, radiologists, and surgeons. The Charlson Comorbidity Index (CCI), which is a measure of burden of disease due to medical comorbidities [43], was calculated for our participants (excluding the current sarcoma). Sociodemographic variables that were studied included gender, age, country of birth, SEIFA score (measure of relative socioeconomic status (SES) based on postcode of residence that is developed and updated by the Australian Bureau of Statistics based on five-yearly census data [44]), being in a partnered relationship, English not being a first language, having had to stop work or recreational activities because of the sarcoma, and the ability to work, drive, partake in leisure activities, and complete activities of daily living (ADL). Tumour-related variables included type of sarcoma, location of sarcoma, surgery, and whether the sarcoma was diagnosed unexpectedly or following a delay.

### 3.2. Psychological Distress and Cognitive Perceptions

The Depression Anxiety and Stress Scale 21 (DASS21) is a 21-item self-report quantitative measure encompassing three subscales of distress:* depression*,* anxiety,* and* stress* [46, 47]. The DASS21 does not assess somatic items and is therefore particularly useful in patients with medical conditions such as chronic pain and malignancies, such as head and neck cancers [27, 48]. Participants are asked to rate 21 items regarding distress according to a 4-point Likert scale (0–3). The final score for each subscale of stress, depression, and anxiety is the cumulative score multiplied by two. Cut-off scores have been developed for each subscale into five categories of severity with higher scores indicating a higher level of symptoms present. Where appropriate, the cohort was dichotomized into two groups according the severity labels from the DASS Manual [45]. The “distressed” group included participants who reported symptoms of distress that categorized them into moderate, severe, or extremely severe categories; the “nondistressed” group comprised patients that reported lower levels of symptoms (consistent with no or only mild distress).

Cancer-related stressors were assessed via free-text to the question “what has been the biggest source of stress, or frustration relating to the cancer journey so far?” with a follow-up telephone call to participants who required clarification of this item.

The Shame and Stigma Scale (SSS) was developed in 2013 by Kissane et al. [49] for use to assess the level of an individual's shame of their appearance, their sense of stigma, regret, and concerns with speech and social interactions. This was developed for use with oral squamous cell carcinoma patients. After discussion with the developer of the tool, a modified version of the SSS was used to assess shame of one's external appearance, following surgery for ES. Participants were asked to rate eight items regarding their attitudes towards the external appearance of their limb on a 5-point Likert scale (0–4). Three items were reverse-coded. The final score was derived by the sum of the five items and the sum of the three reverse-scored items, which was then scaled by conversion to a percentage. A higher SSS score represented a greater sense of disfigurement.

### 3.3. Activity Limitation

The Toronto Extremity Salvage Score (TESS) is an extremity-tumour-specific self-report measure of disability secondary to activity limitation [10]. Ease of completion of 29-30 everyday activities is assessed via a 5-point Likert scale with the option of indicating whether the specific task is not applicable to the respondent. The overall result is represented as a percentage with higher scores indicating better function. There are normative reference values that can be used as a base comparison with the general population [50]. In our study, items have been dichotomized according to difficulty in performing that particular activity, with “caseness” defined as moderate difficulty or harder. This dichotomy has also been used in other studies [51]. The physical function subscale of a non-extremity-tumour-specific QoL tool such as the EORTC QLQ C-30 is not able to discriminate functional status in this specific cancer cohort. As in other QoL studies in ES, QoL tools are therefore assessed in conjunction with a sarcoma-specific functional assessment tool.

### 3.4. Health-Related Quality of Life

The European Organisation for the Research and Treatment of Cancer Quality of Life Questionnaire Core Module 30 (EORTC QLQ C-30) is a cancer-specific, self-report QoL questionnaire. It comprises physical function, cancer symptom, and global health QoL subscales and also assesses financial difficulties. Compared to other global QoL assessment tools, such as the generic Short Form 36 (SF-36), it allows for assessment of symptoms such as nausea, vomiting, and bowel dysfunction [52]. In particular, subjective financial difficulties are assessed, which is especially important in working aged patients with ES. Each subscale ranges in score from 0 to 100; higher scores represent better global QoL and physical function but also higher levels of symptoms (e.g., pain) and more financial difficulties.

## 4. Statistics

The Statistical Package for the Social Sciences (SPSS) version 20.0 (SPSS Inc., Chicago, IL, USA) was used for all statistical analyses and a *P* < 0.05 was considered statistically significant.

Continuous data were expressed as means with standard deviations, percentages, and numbers.

In order to identify the trajectory of distress, we plotted the mean DASS21 scores, the proportion of participants with moderate to severe distress scores, and mean TESS scores at *t* = 0, *t* = 1, and *t* = 2.

A one-way repeated measured analysis of variance (ANOVA) was conducted to evaluate the null hypothesis that there was no change in participant's DASS21 scores when measured at *t* = 0, *t* = 1, and *t* = 2, using the Bonferroni correction to adjust for multiple comparisons.

In order to evaluate person-to-person differences in outcomes, we performed an exploratory data analysis via bivariate analyses. Phi coefficients were used to correlate dichotomized variables whilst Spearman rho correlations were assessed for ordinal and continuous data. Two-tailed values were calculated.

Independent* t*-tests were used to compare means of distressed with nondistressed groups for the null hypothesis that there were no differences in variables studied, including QoL, function, pain, and financial difficulties. We assumed that variances were equal if Levene's test for equality of variances were >0.10.

## 5. Results

### 5.1. Participants

During the recruitment period, 120 patients were referred to the VSS for management of a newly diagnosed ES. Participants were excluded from the study due to cognitive impairment (*n* = 3) and Non-English-speaking background (*n* = 3) or because surgery was not part of planned management (e.g., metastatic disease, palliation, or death) (*n* = 12). Five participants had undergone surgery prior to completing the questionnaires and six patients declined participation for time reasons. 91 participants were finally recruited for our study and completed our baseline questionnaires. Surgery on our participants was performed between August 9, 2011, and January 18, 2013.

76 participants completed the questionnaires at the final timepoint, one year after the index surgery (16.48% drop-out rate, *n* = 15). Six participants had deceased from disease, one participant died from intraoperative complications, one participant developed Alzheimer's disease, one participant moved overseas, and two participants were not contactable and were lost to follow-up. Four participants declined further participation due to time constraints, stating “they were not distressed and wouldn't be helping anybody with their responses anyway.”

The data deviated slightly from a normal distribution; however, the skewness of the comparison groups followed the same directionality and, therefore, independent* t*-tests were carried out to assess between-group differences.

### 5.2. Sociodemographic Data

Characteristics of our cohort are summarised in Table 1. Out of the 76 participants, 40.8% were females (*n* = 31). The ages of our participants in our cohort ranged from sixteen to 86, with a mean of 55.1, a median of 57.0, and a standard deviation of 16.4. Outcome data for each timepoint is recorded in Table 2. Mean TESS scores were 77.40 ± 22.10, 68.83 ± 18.89, and 76.91 ± 18.95; mean DASS21 scores were 21.79 ± 18.60, 25.30 ± 25.62, and 24.09 ± 27.50 at *t* = 0, 1, and 2, respectively.

### 5.3. Objective 1: Source of Stress

Reported source(s) of stress at *t* = 0 are summarized in Table 1. Overall, 30.3% (*n* = 23) of our cohort reported that the disruption to life was the biggest source of stress, whilst 25.0% (*n* = 19) felt the healthcare system was the biggest source of frustration. 11.8% (*n* = 9) of the cohort reported that the loss of independence or change in life roles represented the major stressor and a similar proportion of the cohort worried about the unknown.

### 5.4. Objective 2: Prevalence and Trend of Distress

As summarized in Table 2, the overall prevalence of moderate to severe DASS21 scores for either of the subscales of stress, anxiety, and/or depression was 28.9% for our cohort of recently diagnosed, preoperative patients, 36.8% at six months after surgery, and 31.6% at twelve months after surgery. In terms of the trajectory of distress, of the three subscales of distress, anxiety was most prevalent at baseline (22.4%), but depression was most prevalent after surgery (27.6% at six months and at twelve months after surgery).

**Figure 1:**
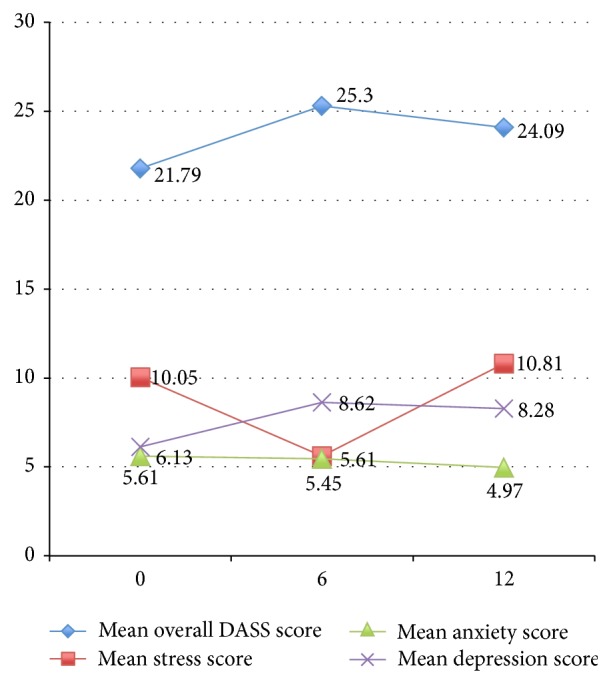
Mean DASS21 score with time

**Figure 2:**
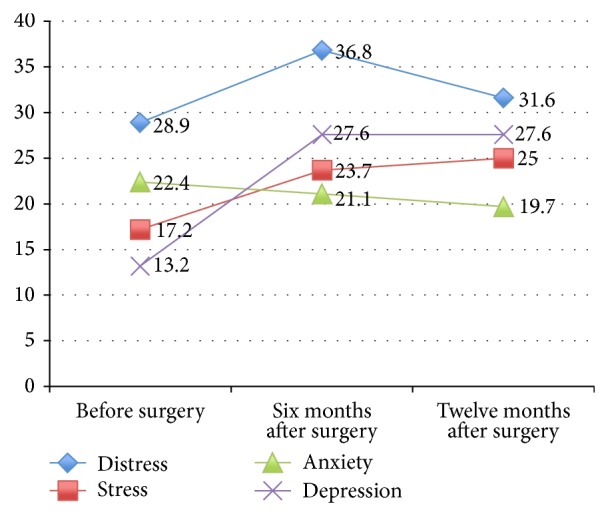
Proportion of moderately to severely distressed participants according to timeframe (based on Table 2).

**Figure 3:**
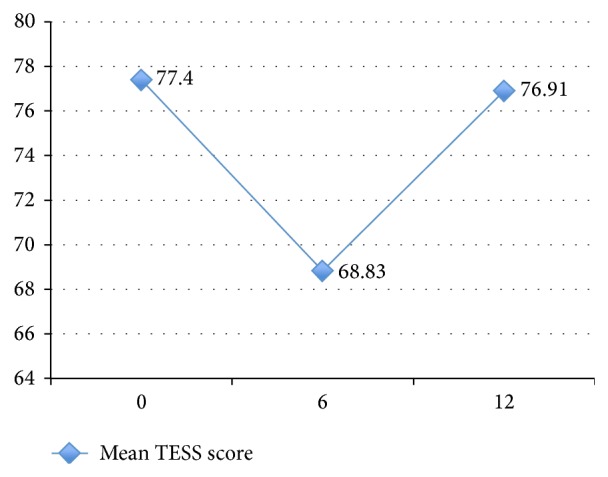
Mean TESS score with time (based on Table 2).

As shown in Figures 1 and 2, mean overall DASS21 scores peaked at six months but reduced at twelve months. Overall, the proportion of our cohort that reported moderate to severe stress and depression scores increased with time, whilst the proportion that reported moderate to severe anxiety scores reduced with time. Distress was most marked at six months. The prevalence of distress according to age showed that older adults were least likely to be distressed, compared to youths, working aged adults, and elderly people. Amongst people aged sixteen to 54 years, fifteen out of 32 (46.9%) were distressed; amongst those aged 55–75 years, only five out of 36 (13.9%) reported significant distress; and amongst those aged 76–86 years, half of the eight elderly people were distressed. TESS scores and ease of completing ADLs appeared to improve with time after surgery (mean TESS at six months was 68.8, which improved to 76.9 at twelve months after surgery) as shown in Figure 3.

Our paired sample* t*-tests showed that there were no significant differences between QoL, mental health, and physical function scores between baseline and twelve months after surgery. However, comparing scores between six months and twelve months after surgery, physical function, role function, and pain scores improved, whilst mental health, overall QoL, and social function scores remained relatively constant (Tables 3(a) and 3(b)).

The results of the ANOVA indicated that there were small and nonsignificant time effects for DASS21 and subscale scores between each of the timepoints. Also, *P* > 0.05, suggesting that DASS21 scores remained relatively stable.

There was a significant time effect for TESS scores, Wilks's Lambda = 0.69,* F*(2, 74) = 17.03, *P* < 0.01, and *η* ^2^ = 0.32. Thus, there was significant evidence to reject the null hypothesis. Follow-up comparisons indicated that there was a significant reduction in TESS scores between *t* = 0 and *t* = 1 and a significant improvement between *t* = 1 and *t* = 2 (both *P* < 0.01) (Figure 3). There was no significant difference between baseline TESS scores and *t* = 2 scores indicating that functional scores had returned to baseline at a year after surgery.

**Figure 4:**
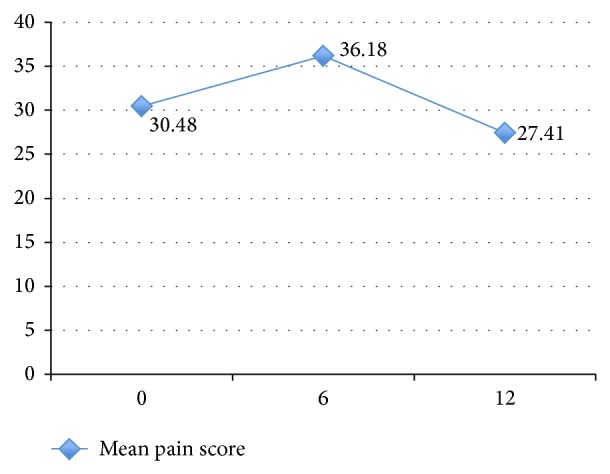
Mean pain score with time.

Pain scores displayed a small to medium, significant time effect, Wilks's Lambda = 0.91,* F*(2, 74) = 3.61, *P* < 0.05, and *η* ^2^ = 0.09, with pairwise comparison indicating that the difference was an improvement in pain scores from *t* = 1 to *t* = 2 (*P* = 0.03) (Figure 4).

### 5.5. Objective 3: Comparing Distressed and Nondistressed Participants

**Figure 5:**
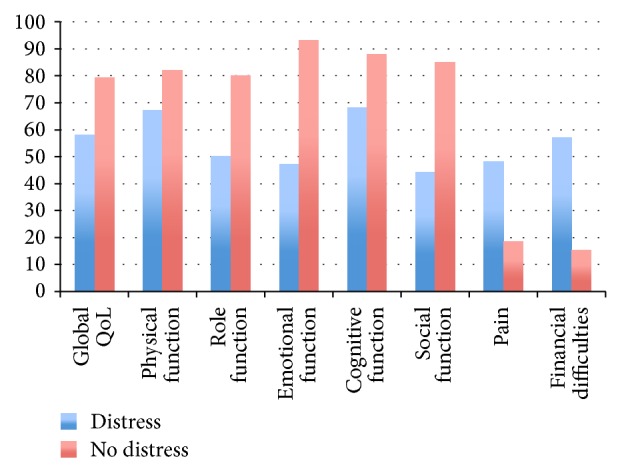
Summary of description of mean scores of EORTC QLQ C-30 (%) domains by presence of distress at 12 months.

Distressed patients had poorer function (MSTS93, TESS) and QoL (from EORTC QLQ C-30) mean scores compared to nondistressed patients as presented in Table 4 and Figure 4. As shown in Figure 5, distressed participants reported worse scores for each domain assessed by the EORTC QLQ C-30.

### 5.6. Objective 4: Correlates of Distress

#### 5.6.1. Baseline and Sociodemographic Variables

DASS21 score at diagnosis strongly correlated with distress twelve months after surgery (*ρ* = 0.421, *P* < 0.01). Reported QoL at baseline was moderately strongly and inversely correlated with distress (*ρ* = −0.35, *P* < 0.01). Being aged between 55 and 75 years was inversely correlated with distress (*ϕ* = −0.361, *P* < 0.01) with moderate strength (*n* = 37, mean DASS21 score = 13.78) whilst being aged younger than 55 years was directly correlated with distress (*ϕ* = 0.28, *P* < 0.05) (*n* = 32, mean DASS21 score = 32.03). DASS21 scores were significantly higher in the younger age group* t*(48.18) = 2.87, *P* = 0.006. Living in a postcode marked as middle SES was moderately strongly correlated with distress (*ϕ* = 0.34, *P* = 0.003) (*n* = 35, mean DASS21 score = 32.14), whilst living in a postcode marked as low SES was weakly inversely correlated with distress (*ϕ* = −0.23, *P* < 0.05) (*n* = 17, mean DASS21 score = 14.82). Distress was higher for people in middle SES compared to people in lower SES* t*(44.22) = 2.46, *P* = 0.02. There was no significant correlation between distress and living in a postcode marked as high SES. Significant pain as a presenting feature and having undergone chemotherapy were weakly correlated with distress (*ϕ* = 0.29 and *ϕ* = 0.23, resp., both *P* < 0.05). We did not find strong, significant correlations between distress and other tumour and demographic variables.

#### 5.6.2. Outcome Variables at Twelve Months

Poor physical function was highly correlated with distress (TESS *ρ* = −0.53, PF *ρ* = −0.49, and RF *ρ* = −0.54). Social functioning and overall QoL were also highly correlated with distress (*ρ* = −0.68 and −0.62, resp.). Pain was also strongly correlated with distress (*ρ* = 0.42) as were financial difficulties (*ρ* = 0.53) and high shame and stigma scores (*ρ* = 0.46). The EF subscale and DASS21 scores were highly correlated (*ρ* = −0.85), indicating that they were both measuring psychological morbidity. All variables were significant at *P* < 0.001.

## 6. Discussion

We report on a cohort study of people with nonmetastatic ES, examining psychological distress up to one year after surgery. Our cohort was heterogeneous in terms of histological subtype and anatomical location of ES but homogeneous for timepoint of the cancer journey, as all participants represented prospectively recruited consecutive patients from an adult cancer institution, with a new diagnosis of ES. All patients received a standard protocol of treatment by a unified multidisciplinary sarcoma service (VSS).

Compared to other longitudinal psychosocial studies on adult ES patients, our cohort consisted of a relatively large sample size with 76 participants. Participants comprised people across a wide range of age groups. When compared to patients suffering from other cancers, a majority of our cohort were still of working age. Sarcoma is a nondiscriminatory cancer in terms of age and it has been reported that 60% of all sarcomas occur in people who are younger than 55 years [53].

### 6.1. Prevalence and Trend of Distress in People with Extremity Sarcoma

About a third of our cohort of people with recently diagnosed ES reported moderate to severe levels of psychological distress at any one point. This is comparable with previous reports on prevalence of distress in general cancer cohorts that included common primaries, including breast and prostate cancers [19, 25, 26, 30]. Our rates of distress appeared to be lower than the rate of distress in head, neck, lung, and pancreatic cancers [18, 19].

Paredes et al. [14] performed a cross-sectional study on sarcoma patients who were grouped according to phase of cancer journey:* diagnosis phase* (*n* = 42),* treatment phase* (*n* = 37), and* follow-up phase* (*n* = 63, mean time of 52.93 months after initial diagnosis). They found that moderate to severe anxiety was most prevalent in the diagnosis phase (29.3%), followed by treatment phase (25%) and follow-up phase (21%). They found that moderate to severe depression was most prevalent in the treatment phase (19.4%) followed by diagnosis phase (19%) and follow-up phase (6.5%). Females and older patients displayed higher levels of depression, whilst recurrence was associated with both anxiety and depression. Anxiety and depression scores reduced with time, reflecting that emotional distress is usually transitory and allows for adjustment.

In our cohort, although repeated measures ANOVA did not find a significant difference between DASS21 scores with time, a similar trend was found as shown in Figure 1. Mean overall distress, stress, and depression scores were most marked after the surgery (six months: 25.3, 11.24, and 8.62, resp.) and reduced with time (twelve months: 24.09, 10.84, and 8.28, resp.). The mean anxiety score was highest during diagnosis (5.61) and waned with time (six months: 5.45, twelve months: 4.97). Mean stress scores at twelve months (10.84) were higher than scores at baseline (10.05). The DASS Manual describes “stress” as being factorially distinct from depression and anxiety and a state that is characterized by nervous tension, difficulty relaxing, and irritability, which is similar to the DSM-IV diagnosis of generalized anxiety disorder (GAD) [45]. This was an interesting finding and perhaps reflected the accumulating incomplete tasks and responsibilities patients accrued that were unable to be fulfilled due to convalescing from the sarcoma and surgery. Rehabilitation following limb salvage surgery takes at least six to eight weeks to restore independent function and much longer if complications arise and if adjuvant therapy is required [54]. There are other possible explanations for the higher stress levels after surgery. It may reflect difficulty relaxing due to instructions on the need to protect the reconstructed limb, to prevent late complications, such as fracture, dislocation, or infection. Furthermore, survivors may report higher stress levels due to concerns about recurrence of their cancer. Unlike other cancers, the aetiology of sarcoma is usually sporadic. “Body betrayal” is the perceived notion that the body or a part of the body has betrayed the self because of illness, such as cancer or disability, despite living a life that did not involve lifestyle risk factors for disease (or, conversely, involved living a life that involved health-promoting behaviours) [55, 56]. This is associated with cancers that are not associated with lifestyle factors and with cancers that result in disabilities, such as breast cancer [57]. Body betrayal may be particularly relevant to ES survivors and may result in negative body image [58]. Survivors of ES may also feel anxious that they cannot do anything to reduce the likelihood of recurrence, which may contribute to a sense of helplessness regarding their future [59].

It would be of interest for future investigations to follow up on the trend of stress and anxiety to evaluate the trend of stress levels further down the track, as well as to conduct a qualitative study exploring the sources of stress in survivors of ES.

### 6.2. Differences between Distressed and Nondistressed Participants and Correlates of Distress

Compared to nondistressed participants, people that were distressed displayed poorer physical function, higher levels of shame, and poorer QoL.

#### 6.2.1. Sociodemographic Variables

Our study has found that AYAs and midlife adults (aged sixteen to 54 years) were more likely to be distressed at one year after surgery for ES compared to people aged 55 to 75 years. This could perhaps reflect that older adulthood is a relatively stable phase of life in terms of career and family responsibilities. Conversely, this could reflect the increased vulnerability of AYAs (defined as people between the ages of fifteen and 29 years) to psychological distress. Adolescence represents a period of developmental transitions, characterised by cognitive, biological, and socioeconomic challenges [60–62]. Health problems such as cancer, in this age group, are uncommon as cancer is predominantly a disease of the older population. Cancer occurring in AYAs represents a disruption in a phase of development, which includes increased responsibility for the self, autonomy in decision making, financial independence, and identity formation [61] and is associated with more psychological distress and lower self-esteem when compared to children and older adults with cancer [63]. It is also recognised that the prevalence of nonadherence to medical advice in the adolescent oncology population is higher than in nonadolescent populations [64]. Paediatric and adult cancer centres may not be adequately equipped to manage the unique demands of cancer patients in this age group [65]. Furthermore, research in other cancer types has shown an inverse relationship between age and unmet needs in the fact that young people with cancer have reported more unmet needs and less satisfaction with the care they received than older people with cancer [21, 66, 67].

Low income, lower educational attainment, and being from an ethnic minority have been found to be significant risk factors for depression and psychological distress in survivors of cancer [2, 19, 29, 37]. Low income is associated with unemployment and financial stress, which in turn is associated with lower QoL and distress [38]. Other risk factors for distress in the psychooncology population that have been described in the literature include female gender [17, 39], younger age [17, 18, 40–42], and poor perceived social support [26, 29, 32].

In our study, we found that financial stress was strongly correlated with distress. However, low educational attainment did not show a significant correlation with distress. Distress was higher for people living in a middle SES area compared to people in a lower SES area. There was no significant correlation between people in a high SES and distress. This may be attributed to people in a high SES having more resources available and having fewer stressors, whilst conversely people in a low SES may have fewer encumbrances, have lower expectations, or be identified as requiring more help and have more access to healthcare services and therefore were less likely to be distressed.

Tumour-related factors were not strongly correlated with distress at twelve months. In breast cancer cohorts, a similar finding of a lack of correlation between cancer variables, such as stage of cancer, and depression exists [29]. This perhaps suggests that a cancer diagnosis presents as an absolute threat to one's mortality and sense of self, which may affect mental health, as opposed to a relative threat based on the relative risk to mortality.

#### 6.2.2. Physical Function

Poor function outcomes, such as low TESS scores and poor physical, role, and social functioning, showed strong correlations with distress. Performance status and physical impairment have been consistently found to be significantly associated with distress in cancer patients and survivors [2, 26, 30–32]. Psychological distress may impair the rehabilitation process that is critical postoperatively to train compensatory muscles to achieve effective gait restoration [68]. From the general orthopaedic literature, it is recognized that depression and anxiety during rehabilitation for orthopaedic conditions have a negative impact on recovery [69] and are associated with poor function and pain outcomes following joint arthroplasty in particular [70, 71]. Furthermore, psychosocial interventions have been shown to improve the effectiveness of rehabilitation after orthopaedic injuries [69]. Conversely, poor TESS scores may result in distress as mediated by poorer role and social functioning.

#### 6.2.3. Shame

Body image is defined as the subjective concept of one's physical appearance based on self-observation and the reactions of others, which may be moderated by patient biological, psychological, and social/environmental factors [72]. Body image disturbance is associated with disfigurement [73], such as amputation, the presence of a visible scar, or an abnormal gait pattern. Maladaptation to a disturbed body image results in shame, which has been described as an affective state in which a sense of disgrace, dishonor, or humiliation may generate a desire to cover oneself [49]. Research on head and neck cancer patients suggests that body image disturbance is associated with mood disturbance, psychological distress, impaired social interactions, and poorer reported QoL [49, 72]. There is limited research regarding body image following ES surgery. Drawing from the research performed on traumatic amputees, poor physical function and pain were found to be associated with body image disturbance [74], which in turn was associated with psychological distress, low self-esteem, social avoidance, depression, and anxiety [74–77]. Adolescents with cancer may be particularly vulnerable to body image disturbance [73, 78] and there may also be a gender difference with regard to the perception of body image [72]. Preoperatively, only a minority of respondents indicated that cosmetic considerations were a major stressor. However, at one year after surgery, we found that higher modified SSS scores correlated strongly with distress, and participants that were distressed were more likely to report higher levels of shame towards their physical appearance compared to nondistressed participants. Negative self-perception of body image may be amenable to interventions that aim to promote resilience and improve self-esteem and social confidence such as through cognitive behavioural therapy and social skills training [77].

#### 6.2.4. Quality of Life and Pain

Distressed participants were more likely than nondistressed cohorts to report poorer QoL in each subscale of the EORTC QLQ C-30, including poorer social functioning, lower cognitive functioning, higher subjective pain levels, and more financial difficulties.

The prevalence of pain in our cohort at twelve months after surgery was 46.1%, whilst the prevalence of moderate to severe pain at twelve months was 19.7%. This rate is consistent with other studies that have found the prevalence of pain in nonterminal and non-head-and-neck cancer patients (e.g., breast, nongynaecological urogenital and lung) to be around 30–50% [79–81]. A cross-sectional study on 149 sarcoma outpatients of varying histological subtypes and stages of disease and at varying timeframes of the cancer journey found that the prevalence of pain was 53% in their cohort. They found that 25% of the patients reported significant pain, 18.1% reported mild pain, 18.8% reported moderate pain, and 16.1% reported severe pain using the Visual Analogue Scale [82].

Distressed participants were more likely to report higher pain scores, and pain scores on the EORTC QLQ C-30 were strongly correlated with distress. Pain directly and indirectly negatively influences mental well-being and QoL [83] and is associated with decreased levels of social activities and social support [33]. Pain is complex and multidimensional in nature and the perception of pain is influenced by psychological distress [84]. It is well recognised that pain is a significant risk factor for psychiatric morbidity in the psychooncology population [17, 25, 33–36]. Malignant bone pain presents with a unique pain state that may be difficult to manage and require multimodal analgesics [85]. Effective pain management is therefore one of the designated goals of cancer care in the Victorian Cancer Action Plan [86]. Furthermore, clinical features of the cancer and adverse effects of treatment may overlap with symptoms of psychiatric distress. Severe persistent pain (more than three months after surgery) following elective joint arthroplasty occurs in about 7–20% and 2–8% of knee and hip replacement patients, respectively, and is predicted by the presence of depression [87]. Although the reason for pain in our cancer cohort was not assessed, it may be due to multifactorial reasons, such as surgical factors, previous tissue damage due to cancer or adjuvant therapy, ongoing tissue damage due to the cancer, and patient factors, such as depression. Pain acts as a stressor, contributing to psychological distress, and conversely being distressed negatively influences an individual's ability to cope with a noxious stimulus. It is clear that pain following ES surgery needs to be managed proactively via a multidisciplinary team, comprising pain specialists, oncologists, surgeons, and mental health professionals.

#### 6.2.5. Distress at Baseline

There is consistent research to suggest that patients with high distress scores or poorer emotional functioning at baseline and those with a past history of a psychiatric disorder are at increased risk of persistent distress in the later parts of the cancer journey [15, 17, 25–29]. We found that distress at baseline was strongly correlated with distress at twelve months after surgery. However, we did not find a significant correlation between a past history of a mental health disorder (depression or anxiety) and distress at twelve months, suggesting that previous psychiatric morbidity per se may not be associated with an increased vulnerability to psychological distress in cancer.

### 6.3. Stressors

Almost a quarter of the cohort reported that the biggest source of stress was consequent upon their perceived frustration with the healthcare system. A mixed quantitative and qualitatively designed study on 295 mixed cancer patients throughout different phases of their cancer journey was performed in the United Kingdom, exploring the unmet needs of cancer patients [88]. They found that the majority of participants rated issues relating to the healthcare system as most important. These included issues relating to confidence in the health professionals, communication issues (such as taking the time to discuss issues with the individual honestly, sensitively, accurately, and respectfully), and easy and quick access to health professionals and health services. In our present study, this largely related to the perception that there was a lack of communication between auxiliary hospital staff (e.g., waiting times for imaging, biopsy, and follow-up appointments) and patients. This is an important area for clinical services to address.

Sarcoma is rare and the clinical features of soft tissue sarcoma may be insidious. Almost half of our cohort reported that their initial diagnosis was delayed due to being dismissed by themselves or a health practitioner, whilst a similar proportion had undergone surgery for a presumed benign condition prior to the diagnosis. Delayed diagnosis may result in tumour progression resulting in higher stage at time of diagnosis. Osteosarcomas that are diagnosed late or unexpectedly are more likely to have amputations and are associated with poorer survival and other worse oncological outcomes compared to osteosarcoma managed efficiently by a sarcoma service [89–91]. Unplanned manipulation of the affected limb such as through poorly planned biopsies and unintentional surgical excision due to misdiagnosis may result in contamination of compartments and require much bigger surgery, including amputation, to remove the tract as a site of seeding of the cancer [89, 92, 93].

This may have implications on the cosmetic outlook and physical function of the patients. This may also have implications on the patient's trust of medical professionals and result in reduced perception and expectation of the healthcare system and influence expectations of recovery. Increased education of primary healthcare physicians regarding identification of potential red flags for sarcoma is necessary in order to reduce the incidence of sarcomas that are diagnosed through unplanned manipulations.

Another commonly reported stressor was the fear of the unknown, in terms of both the logistic/practical aspects of their care and the unexpectedness of outcomes. Previous studies have found that clinical uncertainty is associated with hopelessness and consequent psychological distress [94]. Uncertain expectations have been found to be negatively associated with outcome following extremity sarcoma surgery [13]. Improved communication between hospital and patients may be necessary with a readily contactable liaison person in our integrated sarcoma service. There may also be a role for peer support and psychoeducational therapy to address uncertain expectations, in order to improve outcomes in ES management.

## 7. Implications for Further Research and Limitations of Our Study

Psychological distress is associated with poor outcomes such as poorer QoL and poorer physical function. Distress early on in the cancer journey predicts persistent distress at one year after surgery. Stressors have been identified that may be amenable to psychoeducational interventions. There is great scope for future research to investigate the role of early intervention to improve outcomes in people diagnosed with ES.

As we have discussed above, compared to other cancers, ES affects people of working age. Impaired social functioning and cognition may result in difficulties with work and employment, resulting in higher financial difficulties. Our study found that two-thirds of our cohort had reported that they had to modify or stop their work as a consequence of the sarcoma, and this change in employment status was instituted early on during the diagnosis. More than a quarter of our patients reported that they had persisting significant difficulties with performing their usual work one year after the surgery. Financial stress may be a stressor for distress. Clearly, further dedicated research into employment outcomes in people with ES would be of interest.

One of the limitations in our study included the use of the modified SSS, to assess shame, which is not validated for use in this cohort. As discussed above, body image is an important outcome of interest following ES surgery. Further studies may be required to develop an ES-specific tool to assess body image and to investigate the role of psychological interventions to target negative self-image. Furthermore, our analysis was limited by our sample size. Future studies with larger samples would improve sampling and reduce bias and variability and allow for more robust prediction analyses to be undertaken.

A large proportion of our cohort reported that their ES was either initially dismissed as benign and ignored or diagnosed through surgery for a presumed benign condition. As discussed above, this may be associated with greater morbidity and mortality. Increased education for primary care physicians and general and plastic surgeons, such as a protocol for management of large lumps, may reduce the incidence of unexpected and delayed diagnoses.

## 8. Conclusion

Almost a third of our cohort of recently diagnosed ES patients reported moderate, severe, or extremely severe stress, anxiety, or depression. From previous research, psychological distress is associated with negative outcomes in cancer and distress and may be amenable to management. In our cohort, distress was associated with poor QoL, financial difficulties, pain, and poor physical function. Patients with a past history of depression and/or anxiety and those that are distressed early in the diagnosis phase of the cancer journey may be especially vulnerable to persistent distress up to a year after surgery. In order to optimize outcomes in ES, it is important to actively screen and effectively manage psychological distress.

## Figures and Tables

**Table 1:** Sociodemographic details.

Age (years)		16–86 (mean: 55.1 ± 16.4)

	*N* = 76	%

*Demographic *		
Gender		
Female	31	40.8
SEIFA stratification		
Low	17	22.4
Middle	35	46.1
High	24	31.6
Age group		
16–54	32	42.1
55–75	36	47.4
≥ 76	8	10.5
Country of birth		
Australia	60	78.9
Rurality		
Metropolitan	33	43.4
Regional	24	31.6
Rural	19	25.0
Non-Victorian resident		
Interstate	13	17.1
Education		
Primary	9	11.8
Secondary	36	47.4
Vocational	12	15.8
Tertiary/postgraduate	18	23.7
*Social information *		
Occupation		
Manager/admin/professional	23	30.3
Tradesperson	6	7.9
Sales/personnel/clerks	10	13.2
Machine operator/labourer	8	10.5
Home duties/student	4	5.2
Retired	25	32.9
Marital status		
Partnered: married/de facto	58	76.3
English 1st language		
Yes	69	90.8
Charlson Comorbidity Index		Mean: 2 ± 2.24
*Health information *		
Significant pain as a presenting feature		
Yes	26	34.2
Past history of depression and/or anxiety (no other psychiatric diagnoses listed)		
Depression	15	17.9
Anxiety	3	3.6
Depression and anxiety	1	1.2
Required psychological intervention for cancer diagnosis induced distress on diagnosis		
Yes	13	15.5
*Cancer characteristics *		
Type of sarcoma		
Osteosarcoma, Ewing's sarcoma	9	11.9
Chondrosarcoma	7	9.2
Soft tissue sarcoma	60	78.9
Location of cancer (lower limb *n* = 56, 73.7%)		
Proximal upper limb	11	14.5
Distal upper limb	9	11.8
Proximal lower limb	39	51.3
Distal lower limb	17	22.4
Dominant upper limb involved (out of *n* = 20)		
Yes	9	45
Neoadjuvant therapy		
Radiation only	51	67.1
Chemotherapy	8	10.5
Sarcoma diagnosis delayed: for example, not thinking the lump was anything to worry about; doctor dismissed lump as not important		
Yes	37	48.7
Sarcoma diagnosis made from unplanned manipulation for misdiagnosed benign condition		
Yes	35	44.7
*Surgery details *		
Type of surgery		
Soft tissue resection	56	73.7
Limb-sparing surgery	12	15.8
Amputation	8	10.5
Perception of most significant source of stress at *t* = 0		
Mortality	2	2.6
Disruption to life/plans/goals, for example, career, school, and recreational/social interactions; financial implications	23	30.3
Change in life role(s), for instance, feeling useless, burden on family	9	11.8
Fear of the unknown	9	11.8
Loss of locus of control, for instance, having to wait for things to happen	2	2.6
Cosmetic related concerns	3	3.9
Let down by healthcare system	19	25.0
Other or cannot say	1	1.3
Not stressed/no complaint	8	10.6

**Table 2:** Prevalence of caseness for TESS, DASS21, and EORTC QLQ C-30.

	Diagnosis *n* (%)	6 months *n* (%)	12 months *n* (%)
TESS^*^			
Difficulty performing ADLs	25 (32.9)	34 (44.7)	21 (21.6)
Self-rate as disabled	11 (14.5)	22 (28.9)	16 (21.1)
DASS21^**^	22 (28.9)	28 (36.8)	24 (31.6)
Stress	13 (17.1)	18 (23.7)	19 (25.0)
Anxiety	17 (22.4)	16 (21.1)	15 (19.7)
Depression	10 (13.2)	21 (27.6)	21 (27.6)
EORTC QLQ C-30^***^			
QoL	20 (26.3)	21 (27.6)	19 (25.0)
PF	7 (9.2)	9 (11.8)	3 (3.9)
RF	18 (23.7)	27 (35.5)	14 (18.4)
EF	6 (7.9)	15 (19.7)	10 (13.2)
CF	4 (5.3)	6 (7.9)	6 (7.9)
SF	13 (51.3)	17 (22.4)	13 (17.1)
PA	39 (63.2)^1^	47 (61.8)^1^	35 (46.1)^1^
	22 (28.9)^2^	27 (35.5)^2^	23 (30.3)^2^
	16 (21.1)^3^	18 (23.7)^3^	15 (19.7)^3^
FI	20 (26.3)	21 (27.6)	14 (18.4)

^*^Caseness for TESS defined as moderate difficulty or harder.

^**^Caseness for DASS21 defined as being moderate to severe based on DASS Manual guidelines (S. H. Lovibond and P. F. Lovibond 1995 [45]).

^***^Caseness for EORTC QLQ C-30 subscales: QoL dichotomized at ≤4; other subscales dichotomized as quite a bit or more of difficulty or level of symptoms.

^1^Pain dichotomized at a little or more;^ 2^pain dichotomized at more than a little; ^3^pain dichotomized at moderate to severe.

**(a):** 

	*t*
DASS21	−0.75
TESS	0.18
QoL	−0.92
PF	−0.03
RF	−0.66
EF	−1.47
CF	0.59
SF	0.71
PA	0.71
FI	−0.23

All not significantly different.

**(b):** 

	*t*
DASS21	0.53
TESS	−5.43^*^
QoL	−1.88
PF	−5.21^*^
RF	−5.13^*^
EF	−1.71
CF	−0.34
SF	−1.38
PA	2.66^*^
FI	1.22

^*^ *P* < 0.01 (2-tailed).

**Table 4:** Summary of DASS21, TESS, LOT-R, SSQ, SSS, and 12-month EORTC QLQ C-30 subscale scores (mean ± SD) by presence of distress at 12 months.

	Distress, *n* = 24 (M ± SD)	No distress, *n* = 52 (M ± SD)	Independent *t*-test
DASS21 at baseline	33.6 ± 20.1	16.4 ± 15.2	*t* = −3.74^**^
DASS21 at 6 months	50.0 ± 26.7	13.9 ± 15.1	*t* = −6.18^***^
TESS at baseline	69.9 ± 22.9	80.9 ± 21.1	*t* = 2.05^*^
TESS at 6 months	59.9 ± 19.7	73.0 ± 17.2	*t* = 2.95^**^
TESS at 12 months	66.6 ± 15.3	81.7 ± 18.7	*t* = 3.46^**^
SSS	36.6 ± 21.7	16.8 ± 16.8	*t* = −4.34^***^
Global QoL	57.6 ± 19.6	79.2 ± 18.2	*t* = 4.68^***^
PF	66.9 ± 16.5	81.9 ± 20.4	*t* = 3.15^**^
RF	50.0 ± 33.3	80.1 ± 23.1	*t* = 4.01^***^
EF	46.9 ± 25.2	93.2 ± 10.3	*t* = 8.67^***^
CF	68.1 ± 30.3	88.1 ± 16.9	*t* = 3.04^**^
SF	43.8 ± 28.2	84.6 ± 21.4	*t* = 6.32^***^
PA	47.9 ± 31.6	17.9 ± 25.7	*t* = −4.39^***^
FI	56.9 ± 37.4	15.4 ± 20.3	*t* = −5.11^***^

^*^ *P* < 0.05 (2-tailed).

^**^ *P* < 0.01 (2-tailed).

^***^ *P* < 0.001 (2-tailed).
